# A Scoping Review Mapping Trans* and Gender Diverse People's Representation in Cancer Research

**DOI:** 10.1002/cam4.70774

**Published:** 2025-08-05

**Authors:** Morgan Stirling, Michaela A. Bourque, Mikayla Hunter, John Queenan, Claire Ludwig, Janice Ristock, Lyndsay Harrison, Amanda Ross‐White, Nathan C. Nickel, Annette Schultz, Versha Banerji, Jacqueline Gahagan, Alyson Mahar

**Affiliations:** ^1^ Faculty of Health Sciences, Community Health Sciences University of Manitoba Rady Winnipeg Manitoba Canada; ^2^ Faculty of Health Sciences, College of Nursing University of Manitoba Rady Winnipeg Manitoba Canada; ^3^ School of Nursing Queen's University Kingston Ontario Canada; ^4^ The Ottawa Hospital The University Ottawa Ottawa Ontario Canada; ^5^ Women's and Gender Studies Program University of Manitoba Winnipeg Manitoba Canada; ^6^ Palliative Care Division Bruyère Health Research Institute Ottawa Ontario Canada; ^7^ Queen's University Kingston Ontario Canada; ^8^ Faculty of Health Sciences, Manitoba Centre for Health Policy, Community Health Sciences University of Manitoba Rady Winnipeg Manitoba Canada; ^9^ Faculty of Health Sciences, Max Rady College of Medicine, Internal Medicine University of Manitoba Rady Winnipeg Manitoba Canada; ^10^ Paul Albrechtsen Research Institute CancerCare Manitoba Winnipeg Manitoba Canada; ^11^ Research Office Mount Saint Vincent University Halifax Nova Scotia Canada

**Keywords:** cancer, gender minorities, health equity, scoping review, trans and gender diverse people

## Abstract

**Introduction:**

Trans* and gender diverse people (TGD) are at risk of experiencing inequities across the cancer continuum. While limited evidence suggests cancer is a concern for TGD people, few systematic reviews or other knowledge syntheses exist that can guide efforts to improve the evidence base and address the inequities TGD people face in cancer care.

**Methods:**

Our team conducted a mixed methods scoping review exploring how cancer affects TGD people. We extracted data on cancer type and phase of the cancer continuum, gender definition operationalization, results, and TGD engagement. We followed JBI's meta‐aggregation approach for mixed methods reviews by *qualitizing* quantitative data through narrative interpretation and pooling to integrate the extracted data.

**Results:**

A search of multiple databases yielded 5986 titles after de‐duplication. Reviewers independently screened titles and abstracts and identified 511 citations for full text review, and 55 were included for data extraction. Thirty studies reported on cancer screening, most of which focused on sex‐based cancers. There was significant variation in terminology used to describe TGD people. We observed a lack of breadth in data used among included studies, limiting the generalizability of results. Six studies engaged TGD people. Few studies investigated cancer outcomes or experiences during the diagnosis and survivorship phases; few focused on survival or mortality outcomes.

**Conclusion:**

We observed significant gaps in the body of research on TGD people and cancer. Efforts to improve the evidence base are needed to address knowledge gaps about TGD people's cancer experiences and outcomes and ensure the delivery of inclusive, evidence‐based cancer care is possible.

## 1 Introduction

Trans^1^ and gender diverse (TGD) people are individuals whose gender identity does not align with their sex assigned at birth. TGD people are at high risk of experiencing inequities across the cancer continuum [1, 2, 3]. This includes TGD people having lower screening rates [4, 5, 6], higher incidences of viral‐related cancers (i.e., HPV) [1, 7], and receipt of culturally inappropriate and unsafe care [8, 9, 10]. Connected to this risk is cisnormativity and cissexism [1, 11, 12]. Whereas cisnormativity refers to the pervasive belief that while sex and gender are related but different, they are ultimately concordant with each other and persist across the lifespan [13], cissexism is a related form of discrimination towards those whose gender identity/expression does not or appears to not align with their sex assigned at birth [14].

In the cancer system, cisnormativity and cissexism manifest in a multitude of ways that negatively impact TGD people's cancer outcomes and experiences. For example, TGD people who have changed their sex or gender marker on legal forms may be excluded from organized cancer screening due to these systems' reliance on legal sex to determine eligibility [15]. Cissexism is also apparent in the failure to consider and tailor existing psychosocial supports and resources in ways that can meet TGD people's needs [16]. The consequences of cissexism are profound and result in TGD people experiencing transphobia and discrimination from health care providers [8, 17, 18, 19]. Cissexism also contributes to the failure to prioritize developing inclusive and respectful cancer care models that acknowledge the importance of gender identity and expression in one's cancer journey [8, 9].

Driving TGD people's invisibility in cancer care is a misguided understanding that sex and gender are interchangeable. Conflation of sex and gender is a long‐standing issue that is readily seen in various ways, including within data and health information systems used in cancer research. Table 1 outlines definitions of relevant sex and gender and related terminology. Despite evidence demonstrating the significance of gender in cancer outomes [23], most electronic health records (EHRs) and cancer registries collect information on sex only [15, 24]. When gender variables are present, values more closely align with sex‐related terminology (i.e., female/male). While the landscape related to gender identity terminology continues to grow, few options exist for people to identify outside the binary of male/man and female/woman within EHRs. Those that do often rely on harmful or inappropriate terminology (i.e., hermaphrodite) [25]. The erasure of TGD people in EHRs and related data sources often used in cancer research has profound impacts. Not only does it limit TGD people with cancer's ability to have their identities reflected in their health information and possibly lead to being misgendered by clinicians, but it also limits the capacity to conduct research that can accurately measure and report on TGD cancer outcomes and experiences.

**TABLE 1:** Glossary of terms [20].

Term	Definition
Gender	Social configuration of the roles, behaviors, activities that society typically ascribes to being masculine or feminine [21, 22]
Gender identity	Components of gender that relate to a person's inner self as a woman, man, transgender, nonbinary, etc
Gender expression	Components of gender that relate to one's gendered presentation. Refers to how people express themselves as well as how others perceive them [21]
Sex	Typically refers to an individual's biological processes related to reproductive organs, genes, chromosomes etc. as being male, female, or intersex
Sex‐assigned/designated at birth	Classification of individuals as male or female on the basis of visual anatomy at birth. May be represented as AMAB/AFAB (assigned male/female at birth) or DMAB/DFAB (designated male/female at birth)

Disrupting cissexism and addressing the related inequities TGD people face requires a greater understanding of their experiences as well as their needs. Of importance too are knowledge syntheses that can summarize bodies of literature, identify gaps, and direct action. Few systematic reviews exist, and those that do have focused on specific phases of the cancer continuum (i.e., screening, psychosocial care, survival) [26, 27, 28], included only epidemiologic studies [29], are narrative in nature [30], or only included studies within a limited timeframe [31]. No studies evaluated or reported on available data and terminology describing who is and who is not included.

## 2 Objectives

This review aimed to systematically map the evidence base describing cancer outcomes and explore the literature describing cancer care experiences for TGD adults. This scoping review responded to the broad question of how cancer affects TGD populations through the following objectives: (1) Outline the ways TGD people are described in cancer research; and (2) Describe data sources used to investigate TGD people's cancer outcomes and experiences.

## 3 Methods

### 3.1 Setting and Context

We acknowledge there is a vast array of gender identities and expressions, and it is challenging to use a term that refers singularly to this population's varied and deeply personal experiences of their gender identity/expression. In this study, we use the term TGD in reference to the diverse group of individuals whose gender identity and/or expression does not align with the sex they were assigned at birth, including, but not limited to transgender, non‐binary people and agender people. For conceptual clarity, although many different terms and categories were used to describe TGD people within each study, we will present results using the term TGD. Where necessary, we further categorize TGD people on the basis of sex assigned at birth using the terms AMAB (assigned male at birth) and AFAB (assigned female at birth). We acknowledge this approach may not accurately describe each individual's identity, but suggest that it is a helpful term for underscoring a common experience this population has.

### 3.2 About the Team

Central to this study's goal of mapping and describing TGD people's representation and inclusion in cancer research was an analysis of their relationship to systems of power and oppression, such as health care systems and research more broadly. It also requires an acknowledgment that members of this research team, through their own experiences and relationship to oppression and privilege, may influence the research process. This reflexivity about our own positionality increases this study's transparency and credibility [32].

We are a diverse group of researchers and TGD people with different backgrounds and experiences. Among this team are epidemiologists, clinician‐scientists, health services researchers, critical scholars, nurses, and trainees. Some members of the team identify as part of the TGD population, and some identify as allies. As a team and as individuals, we are committed to doing research that can facilitate systemic change to address inequities TGD and other underserved populations experience in the cancer system.

### 3.3 Design

The scoping review followed a framework that was initially developed by Arksey and O'Malley, and expanded upon by Levac et al., Colquhoun et al., and Peters et al. [33, 34, 35, 36]. This established approach included the following steps: identifying the research question; identifying relevant studies; selecting studies; charting the data; collating, summarizing, and reporting results; consulting with relevant stakeholders; analyzing evidence; presenting results; and noting implications within findings. We anticipated a variety of qualitative and quantitative studies to be included in this review, and as such, a priori determined to adapt the Joanna Briggs Institute guide for Mixed Methods Systematic Reviews and Meta‐Analyses to align with a scoping review. Results of this scoping review adhere to the Preferred Reporting Items for Systematic Reviews and Meta‐Analyses extension for Scoping Reviews checklist and explanation [37].

### 3.4 Search Strategy and Information Sources

The primary search strategy was developed for Ovid Medline by the research team in collaboration with a librarian (ARW) and modified for the other databases. Full search strategies are provided in the Supporting Information. We executed similar searches in Embase, Cochrane, and PsycInfo (Ovid), CINAHL, LGBQT+ Life (EbscoHost) and Scopus. Terms for the TGD population were based on previous searches done by the University of Alberta and the University of Minnesota researchers, as well as additional work by Thelwell et al. [38]. The initial search was conducted on September 13, 2022, with follow‐up searches run from September 15, 2022, through November 15, 2022 (see supplemental data for exact dates).

### 3.5 Evidence Screening and Selection

Following the search, all identified citations were uploaded into Covidence and duplicates were removed. The inclusion and exclusion criteria for studies followed the population, concept, and context categories for scoping reviews [39]. We included studies that focused on TGD people who had cancer experience either as a patient or caregiver, were published, peer‐reviewed, written in English or French, and used primary data. We included studies that described experiences and/or outcomes along the following cancer continuum phases: risk, screening, diagnosis, treatment, survival, survivorship, and mortality. We included qualitative, quantitative, and mixed methods studies. We excluded opinion papers, editorials, case reports, reviews, theses/dissertations, and conference abstracts. The full inclusion/exclusion criteria can be viewed in the study protocol [39].

We piloted the inclusion/exclusion criteria on a subset of citations to ensure consistency. An initial screen of titles and abstracts was completed, followed by reviewers screening full‐text articles for inclusion. Conflicts were resolved through discussion. Upon completing the initial full‐text screen, the team decided to separate full‐text articles into three groups: those focused on TGD people, those focused on sexual minorities, and those focused on both TGD people and sexual minorities. This review reports results from studies focusing on TGD people and studies that included both TGD people and sexual minorities; however, only studies that separately reported results for TGD were included.

### 3.6 Charting the Data

A data extraction chart was developed by the research team that was then piloted on a subset of studies to ensure consistency. MS, MB, and JQ completed data abstraction on all studies. We abstracted data related to study characteristics (year of publication, country of study) as well as study design and data source. We abstracted cancer‐related characteristics of the study (e.g., type of cancer and point along the continuum), study results as well as characteristics related to the TGD population (how gender diversity was operationalized, gender diversity measure, and which TGD identities were included in the study). We also abstracted data related to whether studies reported engaging TGD people in the study.

### 3.7 Collating, Summarizing, and Reporting the Findings

A key feature of a mixed methods scoping review is integrating qualitative and quantitative results to provide a comprehensive overview of the phenomenon being investigated [40]. This review followed JBI's meta‐aggregation approach for integrating data in a mixed methods review [40]. For this review, we employed a convergent integrated approach to synthesize and integrate results, which involves abstracting qualitative and quantitative studies simultaneously. The following step was to convert abstracted data into a mutually compatible format, which was accomplished by *qualitizing* the quantitative data that transforms numerical results into a textual description so that they can be integrated with the qualitative data. This is the recommended approach as it is less error‐prone than attributing numerical values to qualitative data [40]. To integrate results, we pooled qualitized and qualitative data together by categorizing results based on which a priori defined study objective (i.e., objective 1 or 2) pooled results related to. We further refined categorized results by systematically identifying similarities and differences and then narratively summarized them to respond to each study objective.

### 3.8 Patient and Public Engagement

A community advisory board comprising 11 TGD people informed the development of the scoping review study design and this manuscript. Advisory board members identify as a trans* and/or gender‐diverse person and have a range of cancer experiences, which include experience as a cancer patient, a caregiver to someone with cancer, or participating in cancer screening. This advisory board is currently collaborating with a larger research team on a project related to TGD people in cancer health services research. Advisors were recruited through advertising on social media, referrals from interested folks, as well as using the larger research team's networks. Two advisors were involved in developing the study protocol by providing suggestions about which data to focus on abstracting and providing feedback [39]. Study results were presented to the advisory board for feedback. Advisors were specifically asked to provide their perspectives on how to acknowledge the numerous identities that are part of the TGD community, which were represented in the literature included in this review, as well as TGD people whose identities were not named. Advisors were also asked to share their perspectives about which results to prioritize. Advisors emphasized the importance of reporting on the ways TGD people are categorized, measured, and represented in the manuscript. Advisors also described the significance of reporting on the engagement of TGD people in cancer research. Advisors were offered the opportunity to review the draft manuscript and provide feedback.

## 4 Results

**FIGURE 1:**
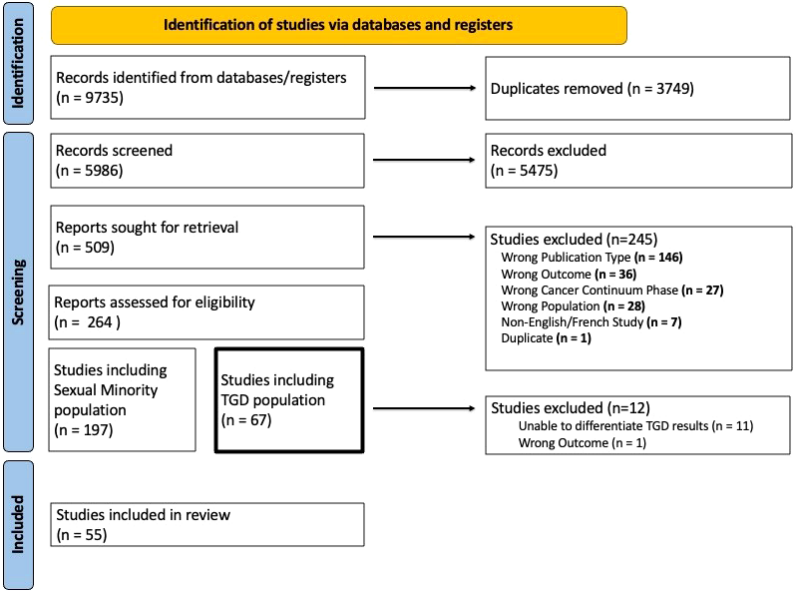
Study flow chart.

The literature search yielded a total of 9735 articles (Figure 1). Of these, 67 articles reported results for TGD people, and 55 articles met all eligibility criteria and were included in this scoping review [4, 7, 8, 18, 19, 41, 42, 43, 44, 45, 46, 47, 48, 49, 50, 51, 52, 53, 54, 55, 56, 57, 58, 59, 60, 61, 62, 63, 64, 65, 66, 67, 68, 69, 70, 71, 72, 73, 74, 75, 76, 77, 78, 79, 80, 81, 82, 83, 84, 85, 86, 87, 88, 89, 90, 91].

## 5 Study Characteristics

Study characteristics are summarized in Table 2. Over half of the studies were published in 2020 and later (*n* = 28). Most studies were conducted in the United States (*n* = 38) and two‐thirds of studies (*n* = 35) did not report a study design. Among studies reporting specific designs (*n* = 20), most authors described their design as cross‐sectional (*n* = 8). Other reported designs were cohort (*n* = 4), narrative (*n* = 2), case–control (*n* = 1), convergent parallel (*n* = 1), exploratory (*n* = 1), interpretive description (*n* = 1), phenomenological (*n* = 1), and survey (*n* = 1).

**TABLE 2:** Summary of study characteristics.

	*N*	% of studies	Studies
Year			
2010	1	1.8%	89
2011	1	1.8%	44
2012	0	N/A	N/A
2013	2	3.6%	60,90
2014	2	3.6%	47,80
2015	2	3.6%	45,49
2016	3	5.5%	8,41,64
2017	6	10.9%	73–75,78,83,84
2018	7	12.7%	19,42,50,52,62,76,86
2019	3	5.5%	53,56,67
2020	12	21.8%	18,48,51,57,59,61,63,72,79,82,85,91
2021	9	16.4%	7,43,46,55,58,65,68,71,88
2022	7	12.7%	54,66,69,70,77,81,87
Location			
Australia	4	7.3%	65,66,81,87
Belgium	2	3.6%	89,90
Canada	3	5.5%	8,18,67
El Salvador	1	1.8%	72
Netherlands	5	9.1%	44,55‐57,60
UK	2	3.6%	46,51
USA	38	69.1%	7,18,19,41,43,45,47‐50,52‐54,58,59,61‐64,68‐71,73‐80,82‐86,88,91
Cancer type			
Anal	2	3.6%	58,82
Breast	17	31.0%	8,18,45,49,50,53,56,60,67,70,75,77,82,86,88,89,91
Cervical	24	43.6%	8,18,41,42,46,53,54,59,61,63‐65,67,72,73,77‐79,80,82,83,86,88,91
Colorectal	4	7.3%	53,67,82,86
Lung	3	5.5%	68,82,85
Ovarian	2	3.6%	8,18
Prostate	5	9.1%	57,71,82,86,88
Skin	1	1.8%	82
Uterine	2	3.6%	8,18
Not specific	17	31.0%	7,19,43,44,47,48,52,55,62,65,69,74,76,81,84,87,90
Cancer continuum phase			
Risk	11	20.0%	48,49,56,57,60,62,68,74,76,84,90
Screening	30	54.5%	41,42,45,46,53,54,58,59,61,63‐65,67,69‐73,75,77‐80,82,83,85,86,88,89,91
Diagnosis	2	3.6%	7,68
Treatment	12	21.8%	7,8,18,19,43,50‐52,65,68,81,87
Survival	1	1.8%	68
Mortality	4	7.3%	7,44,47,55
Survivorship	2	3.6%	48,81
Study design			
Case control	1	1.8%	90
Cohort	4	7.3%	44,55–57
Convergent parallel	1	1.8%	64
Cross‐sectional	8	14.5%	46,48,50,67,71,72,82,85
Exploratory	1	1.8%	63
Interpretive description	1	1.8%	43
Narrative	2	3.6%	8,18
Phenomenological	1	1.8%	65
Survey	1	1.8%	51
Not reported	35	63.6%	7,19,41,42,45,47,49,52‐54,58‐62,66,68‐70,73‐81,83,84,86‐89,91

Over half of the studies focused on a single cancer type (*n* = 29). Seventeen were not focused on any specific cancer type. The remaining studies focused on multiple cancers, including studies focusing on sex‐specific cancers (breast, gynecological, and prostate) or screening‐specific cancers (breast, cervical, prostate, anal lung, colorectal, and skin). Among cancer types included, the most common was cervical (*n* = 24), followed by breast (*n* = 17), prostate (*n* = 5), colon/colorectal (*n* = 4), lung (*n* = 3), anal (*n* = 2), ovarian (*n* = 2), and uterine (*n* = 2). One study included skin cancer.

Most studies investigated a single point along the cancer continuum (*n* = 52). Screening, which included participation and adherence, was the most frequently investigated (*n* = 30). Treatment was the second most investigated phase (*n* = 12), followed by risk, defined as cancer incidence (*n* = 11) Four studies investigated mortality, two studies each investigated survivorship and diagnosis, and one study investigated survival.

## 6 Objective 1

Outline the ways TGD people are described in cancer research.

### 6.1 Operationalizing Gender Diversity

There was variation in approaches for conceptualizing and operationalizing gender diversity across the studies. Twenty‐one studies did not include specific language outlining who TGD people were relative to their research study. Among the 34 studies that provided a description, 31 included verbiage outlining gender diverse people as being individuals whose gender identity and/or expression differs from their sex assigned at birth [7, 41, 42, 45, 46, 47, 48, 51, 53, 54, 55, 56, 57, 59, 63, 65, 66, 67, 72, 73, 74, 75, 76, 78, 79, 80, 83, 85, 86, 87, 88]. One study defined gender diverse people as those who “desire, plan to undergo, or have undergone sex change surgery” [68]. Another study defined gender diversity as transexual people being those with “apparently normal somatic sexual differentiation who feel strongly they actually belong to the opposite sex” [60]. A third study reported gender diversity by using the term Trans which “*is used as a stand in place of “sex” or “gender” as a wildcard for a wide range of identities and expressions, further problematizing the binary applications of sex/gender and, additionally, contributing to systems of knowledge that locate the margins of sex/gender as sites of knowledge production*” [8]. Just over half of studies reporting a definition (*n* = 18 or 53%) included an acknowledgement that gender diversity included a variety of gender diverse identities such as trans*, non‐binary, genderqueer, or gender non‐conforming [7, 41, 46, 47, 48, 51, 53, 54, 63, 66, 67, 73, 76, 78, 79, 86, 87, 88].

### 6.2 Terminology Describing Sample Populations

In our review, we observed that while there was significant variation in terminology used to describe TGD samples, studies generally followed two approaches to describe their sample. In the first, authors described their sample using a broad umbrella term such as transgender to categorize all participants, effectively limiting the diversity of identities that could be represented and described in the data. The second approach provided greater granularity and attention to the diversity of TGD identities and involved categorizing the sample into smaller TGD groups that either described the participants as ‘trans’ gender for example, trans men or trans women or referred to people by their sex assigned at birth that is, AMAB or AFAB.

#### 6.2.1 Studies Using Umbrella Approach (*n* = 18)

**FIGURE 2:**
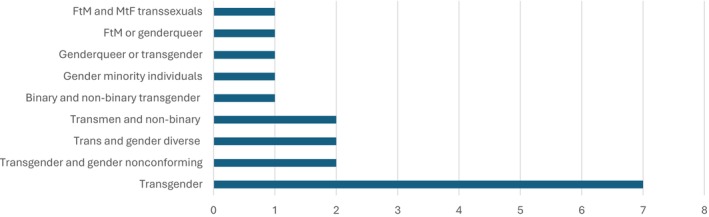
Terminology to describe the sample using the umbrella approach.

Eighteen studies used the umbrella approach (Figure 2).Seven studies described the participants in their sample using the term transgender [7, 43, 47, 62, 76, 85, 88]. One of these studies specified trans as being binary and non‐binary transgender people [81]. Two studies used the terms transgender and gender nonconforming to describe their sample [74, 82]. Two studies used trans and gender diverse as an overarching term to describe all participants in their sample [65, 66]. Two studies reported on transmen and nonbinary people as a single category [46, 51]. One study referred to participants in the sample using the term gender minority individuals [68]. One study described their sample as genderqueer or transgender [50]. One study described TGD people using the category FtM or Genderqueer [64]. One study described the sample using the term transgender; however, it later referenced individuals as FtM and MtF transsexuals [49].

#### 6.2.2 Studies Using Granular Approach (*n* = 37)

**FIGURE 3:**
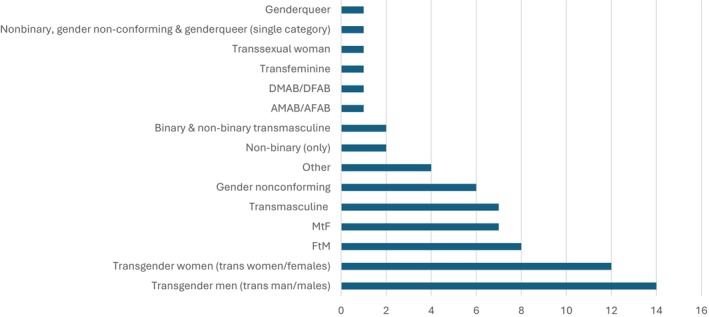
TGD identity‐specific terminology used to describe the sample.

Thirty‐seven studies used a granular approach (Figure 3). Fourteen studies included transgender men (also described as trans man/trans males) as a category [19, 45, 48, 55, 56, 58, 61, 63, 72, 83, 86, 87, 90, 91]. Twelve studies included transgender women (also described as trans women/trans females) as a category [19, 45, 48, 55, 56, 57, 58, 61, 86, 87, 90, 91]. One study referred to participants in their sample as transsexual women [89]. Nine studies categorized participants using transmasculine and/or transfeminine as a way to capture a variety of identities or expressions that include binary transgender people (man/woman) as well as individuals whose identity or expression is masculine/feminine but may not identify as a binary man/woman. Seven studies included transmasculine people [41, 42, 59, 73, 78, 79, 84], two of which further categorized participants into binary and non‐binary transmasculine people [41, 42]. One study reported on transfeminine people [84].

Numerous studies referred to participants in their respective samples by noting sex assigned at birth using the abbreviations MtF [44, 53, 60, 69, 71, 75, 77] and FtM [44, 53, 54, 60, 69, 75, 77, 80]. Two of these studies referred to MtF and FtM [44, 60] participants more generally as transsexual people, while the remaining referred to the larger sample as transgender people. One study used the abbreviation AMAB/AFAB [67]. One study used the AMAB/AFAB abbreviation to specifically categorize gender non‐binary, genderqueer, and gender nonconforming people [91]. One study categorized its sample using the abbreviation DMAB/DFAB (designated male/female at birth) [70].

Seven studies specified the participants in their sample were gender diverse identities and/or expressions typically considered different from trans* people insofar as these people tend to see themselves outside the man/woman binary. Two studies reported on non‐binary people [52, 87], six studies included gender non‐conforming people [8, 18, 48, 53, 75, 86], and one study reported on genderqueer people as a non‐trans identity [18]. In addition, four studies included ‘other’ as a separate category for individuals whose gender identity/expression was not otherwise captured [19, 61, 69, 87].

### 6.3 Approaches for Identifying and Categorizing TGD People

**FIGURE 4:**
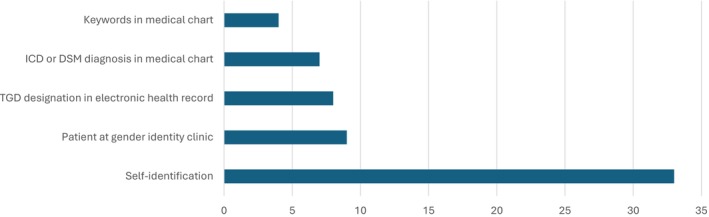
Approaches used to identify TGD people in studies.

Researchers used two broad approaches to identify TGD people in studies (Figure 4). The first approach was for participants to self‐identify as a TGD person (*n* = 33) [8, 18, 19, 41, 42, 43, 46, 48, 50, 51, 52, 53, 58, 63, 64, 65, 66, 69, 71, 72, 73, 74, 75, 77, 78, 79, 81, 82, 83, 85, 86, 87, 88]. The other broad approach was for authors to categorize participants on the basis of what information was available in the data. Nine studies identified TGD people based on whether they were patients at a specialized gender clinic [44, 55, 56, 57, 59, 60, 80, 89, 90]. Numerous studies identified TGD people by the presence of predetermined and fixed categories within existing data. Seven studies relied on the presence of transgender‐related diagnostic ICD or DSM codes in a patient's medical history/chart to identify TGD people [47, 49, 54, 70, 84, 90, 91]. Four studies searched for keywords in a clinical chart or note that was related to TGD people, such as transgender, gender dysphoria, or gender identity disorder [62, 70, 84, 91]. Eight studies identified TGD people by the presence of a specific TGD designation in EHR/registry data [7, 45, 54, 61, 62, 67, 68, 76]. Two studies explicitly specified that the EHR's value for TGD people was transsexual; however, the authors recognized the complications with this term and instead used transgender when reporting results [7, 76].

## 7 Objective 2

### 7.1 Describe How TGD People's Cancer Outcomes and Experiences Are Investigated

Our review identified that studies of TGD people's cancer outcomes and experiences were limited in the scope of data sources used in this research as well as a significant lack of engagement with TGD people in the completed work. Many studies relied on the same data sources (i.e., same survey respondents, same interviewees, same patients at a particular clinic), and few studies engaged with TGD people in the design or implementation of their research, effectively limiting the range of experiences that constitute the evidence base on TGD people and cancer.

### 7.2 Data Sources

**FIGURE 5:**
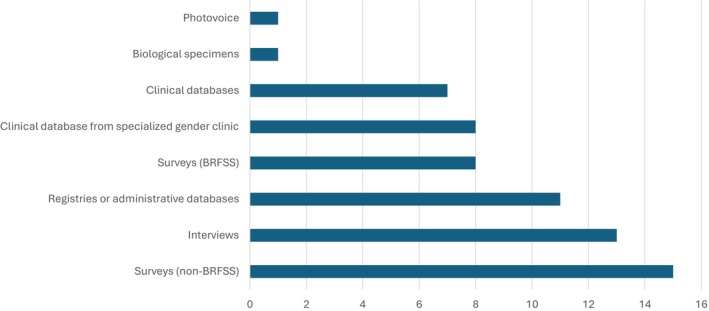
Data sources used in studies.

Different types of data were used across the studies (Figure 5). Data in reviewed studies included information from clinical databases (including EHRs) (*n* = 15) [44, 45, 54, 55, 56, 57, 59, 60, 61, 67, 70, 80, 84, 89, 91]. Among those studies, over half of the clinical databases were reported to come from single‐site specialized gender clinics (*n* = 8) [44, 55, 56, 57, 59, 60, 80, 89]. The remaining clinical database studies were primarily from non‐specialized single sites, except for one study that included data from 3 sites that were part of the same clinical network (Kaiser) [84] and one study that included five federally qualified health centers (USA) [61]. Eleven studies used routinely collected data such as registries (i.e., cancer registry) or administrative databases [7, 44, 47, 49, 55, 56, 57, 62, 68, 76, 84]. Twenty‐three studies used data collected from surveys [19, 42, 46, 48, 50, 51, 53, 58, 64, 66, 69, 71, 73, 74, 75, 77, 81, 82, 83, 85, 86, 87, 90]. Among studies using survey data, one‐third (*n* = 8) used data from the Behavioral Risk Factor Surveillance System between the years 2014–2018 [48, 53, 71, 74, 75, 77, 85, 86]. One survey recruited participants from a specialized gender clinic [90]. Thirteen studies used data collected through interviews [8, 18, 41, 43, 52, 63, 65, 73, 78, 79, 81, 87, 88]. One study used biological specimen data from HPV self‐sampling and [72] and one study used data collected through a photovoice approach [81].

### 7.3 Engagement

There is growing recognition of the importance of engaging affected communities and those with lived experience in cancer research, especially research seeking to address health inequities [92]. In our review, seven studies reported formally engaging TGD people during the research project, and among those that did, the direct role and contributions of TGD people and communities in the research process were often unclear [46, 65, 66, 72, 81, 83, 87]. Of these, three studies referred to consulting and involving the TGD community throughout the project [46, 66, 83]. Two studies reported engaging TGD people through a steering committee; however, these studies were both part of the same larger parent study [81, 87]. One study referred to conducting the project in partnership with a local TGD‐serving organization [72] and one study reported using a TGD reference group, which was not further explained [65].

## 8 Discussion

Our scoping review mapped how TGD representation has been represented in the literature and also methodological limitations with data sources to investigate cancer outcomes and experiences among TGD people. Associated concerns with representation and methodological limitations found within the base of evidence focused on TGD people and the effects of cancer are described below.

Across the included studies there was significant variation in the way TGD people were described within research. While most studies provided a conceptual description that emphasized how TGD people experience a mismatch between their gender identity and/or expression with their sex assigned a birth, several studies described TGD people as individuals who want and/or do undergo some type of medical transition, such as gender‐affirming surgeries to change their sexual anatomy. The emphasis on medical transition as a defining characteristic of TGD people is harmful. Some TGD people undergo some type of medical transition, while some do not [93, 94]. Relying on narratives such as “being born in the wrong body” [95] to define TGD people shrouds the wide spectrum of people who have different relationships between their corporeal body and experienced identities, and excludes TGD people who have or do not wish to not medically transitioned. Moreover, more than one‐third of studies did not provide a description of TGD people. While we note there is ongoing discussion on the best approaches to define and categorize TGD people in research [96, 97], the failure of some studies to include any operational description is problematic. Not only does it lead readers to rely on assumptions and narratives that can flatten the diversity of experiences within this population [97], it requires readers to apply their own understandings of what gender diversity is and who TGD people are which may be inaccurate and negatively impact how they interpret study results and associated implications.

It is important to note that variation and lack of consistency in terminology are a function of what exists in available data sources to complete cancer research, as well as the decisions made by individual researchers and teams in collecting new or using existing data. An outcome of cissexism is that data systems have not prioritized accurately capturing gender and gender diversity, which was observed in this review through studies using limited and sometimes inappropriate categories for TGD people. For example, our review identified the continued presence of the term transsexual in several studies. This term has a complicated history among TGD people, and although it is still used by some people to describe their identity, it is considered derogatory by some TGD people [95]. Historically, we note this term's use may be reflective of an individual's deliberate decision to identify with the term, which may be influenced by cultural contexts or a preference to align their trans identity with practices and desires that require interacting with medical institutions or with legal bureaucracies [95]. However, this is not an experience universally shared by TGD people, and given transsexual people's historical medicalization and pathologizing, it is problematic to use transsexual without some type of acknowledgment and explicit description of who uses it to define their identity [98], Although the vast majority of studies did not use transsexual as a specific group, the use of this term within a small body of literature could have negative implications for TGD people, as clinicians and other researchers reviewing this research may believe it is appropriate for describing all TGD people and inadvertently use it in ways that harm some TGD people.

Due to cissexism and data systems' failure to implement approaches for collecting gender identity data, it is inevitable that many researchers investigating this area rely on data that does not accurately or respectfully describe TGD people. Significantly, lack of accurate data has been identified as a major barrier to conducting high quality cancer research related to TGD people's cancer experiences and outcomes [99, 100, 101]. In this review, we observed a significant number of studies extracting data from EHRs, which are well known to conflate sex with gender or use potentially inappropriate and harmful language like transsexual or hermaphrodite [25, 102]. We also observed that studies relying on diagnostic codes or key words for identifying TGD people may inadvertently serve to reinforce a false narrative that this population has underlying gender‐related medical conditions or exclude those who do not wish to have a TGD‐specific diagnosis [103]. Reliance on inaccurate data may be the most feasible or pragmatic approach for investigating TGD people's cancer experiences or outcomes, but that does not mean researchers should simply accept and reproduce what exists within the data. There are strategies that researchers can employ to challenge cissexism within their work (e.g., Waters [104] and Marshall [105]). An example we found in this review was Nash et al. [76] who identified that the data they used referenced harmful language (i.e., transsexual or hermaphrodite) and instead explicitly described rewording it into terms that are more commonly accepted (i.e., transgender and differences in sex development).

A key strategy researchers should consider is engaging TGD people in their work. We observed that TGD people are not routinely engaged in cancer research about them. Just over 10% of studies (*n* = 7) reported formally engaging TGD people in their studies. The failure to engage TGD people has also been noted in TGD health research generally [106]. There is growing emphasis to increase engagement and participation of individuals with lived experience in research [105, 107]. Organizations such as the World Professional Association for Transgender Health (WPATH) have identified an imperative for researchers to engage TGD people to ensure research is relevant and meaningful to them [105, 108]. Similarly, the Canadian Professional Association for Transgender Health (CPATH) specifies engaging TGD people in research as an overarching principle and outlines key considerations for doing so in appropriate and respectful ways [105, 109]. These organizations, as well as other research, highlight that engaging TGD people in research provides an opportunity to identify the population's research priorities and minimize harm against TGD people [107, 108, 109]. Engaging TGD people in cancer research is a critical step towards disrupting cissexism in the research environment and developing new approaches that are better situated for describing their experiences in meaningful and respectful ways [104].

While these are strategies researchers can employ within their own work, there is also a very clear imperative to improve data systems ability to accurately and meaningfully collect gender identity data. Although there is no consensus on what those changes should look like, at the very minimum, recommendations highlight collecting information on people's gender identity and sex assigned at birth and data needed for billing purposes [110]. A recent article by Pratt‐Chapman et al. further outlines potential options to include for capturing gender identity when collecting data [111]. In addition to the need for changes across data systems, funding bodies such as NIH, NIHR, and CIHR must require TGD engagement in research grant development, advocate for improvements, and provide funding for developing TGD cancer cohorts.

Through the review, we identified significant limitations in the scope of the research on TGD people and cancer. A notable gap in the body of evidence was the absence of randomized controlled trials (RCTs) and prospective cohorts, which are considered high‐quality evidence in evidence‐based practice [112]. A recent study observed that transgender people reported higher rates of participating in clinical trials compared to cisgender people [113]. While these results should be regarded with caution, they do suggest TGD people may be open to participating in clinical trials. Increased emphasis on including TGD people in RCTs is necessary to develop high‐quality clinical practice guidelines that can disrupt cissexism and support oncology clinicians in delivering appropriate, evidence‐informed cancer care.

We further identified a lack of breadth of data sets used in the studies. Almost 15% of studies (8 of 55) used the BRFSS data from overlapping years (2014–2018). Although the BRFSS is an important data source, previous research has highlighted important methodological issues with the BRFSS for TGD health research, including measurement error and misclassification bias attributed to not including non‐binary and gender diverse identities (outside a transgender binary of MtF and FtM) as options for respondents to select [114], as well as interviewers relying on the timbre of respondents' voices to determine sex at birth [115]. While changes were implemented in the 2016 and later versions of BRFSS, reliance on sample weights from earlier versions may negatively impact the ability to generate accurate estimates and result in inaccurate interpretations [114].

In addition to the reliance on the BRFSS, we also noted reliance on several studies accessing data sources from the same or similar samples. This was particularly evident in studies reporting results from specialized gender identity clinics. Notwithstanding how important these clinics are for TGD healthcare or the usefulness of these studies for understanding TGD people's cancer outcomes and experiences, it is necessary to highlight dependence on these samples limits the breadth of who is represented in TGD cancer studies as well as the generalizability of findings to the broader TGD population, as not all TGD people access medical care. Although reliance on these samples is likely somewhat influenced by lack of accurate data elsewhere, it bears emphasizing a reality that these studies cannot represent the diversity of TGD people's cancer experiences and outcomes.

## 9 Limitations

Our study has several limitations for consideration. We did not search gray literature, which may have resulted in excluding published reports by organizations interested in TGD people and cancer. The initial search revealed a significant number of records focused on cancer prevention (e.g., smoking cessation, exercise interventions, etc.), as a team we ultimately decided to exclude these studies as they did not focus on cancer‐specific experiences or outcomes. We excluded studies that discussed the broader 2SLGBTQ+ community in situations where we could not distinguish TGD people in the sample. As TGD people may have similar experiences with cancer care as sexual minority groups, the decision to focus exclusively on studies with a distinct TGD population may inadvertently narrow the results included in this review. We did not include unpublished studies, and so publication bias may have influenced the results of included studies, particularly as studies investigating TGD people and cancer may be underpowered and results may not be considered significant. Countries where TGD identities are not legal would not be represented, and these individuals may be at the greatest risk of experiencing negative cancer outcomes and experiences.

## 10 Conclusion

Our scoping review has systematically investigated and mapped the literature examining cancer outcomes and experiences for TGD people across the cancer continuum. We observed a significant degree of variation in the way gender diversity was operationalized as well as which TGD groups were included within these studies. We also identified a lack of high‐quality data, which is fundamentally connected to a cissexist environment that has not prioritized establishing safe and inclusive processes for collecting and using gender diversity data. Compounding this issue is the reality that so few studies engaged TGD people in their research. Improvements in data collection and use, as well as more intentional efforts to engage TGD people are important mechanisms for strengthening the evidence base. Doing so will also facilitate broadening the scope of cancer research and enable prioritization of research in non‐screening‐related cancer phases.

Numerous structural and behavioral changes are required to improve the research environment wherein TGD people's cancer care experiences and outcomes are studied. Chief among those changes is modifying processes for how cancer registries collect and report gender to include dynamic and inclusive measures of gender diversity. Cancer researchers must also meaningfully consider and integrate TGD people into research programs and studies. In the absence of accurate data, researchers should review best practices for measuring and reporting on TGD people in health research. Addressing research gaps further requires funding agencies to prioritize cancer as a health issue for TGD people and provide targeted funding opportunities to improve cancer research, cancer care, and cancer outcomes.

## Author Contributions

M.S. and A.M. conceived and designed the study; A.R.W. designed and implemented the search strategy; M.S., M.A.B., and M.H. reviewed studies for inclusion, and M.S., M.A.B., and J.Q. extracted data; M.S. led manuscript development and wrote the initial draft, with A.M. providing support; M.A.B., M.H., J.Q., C.L., J.R., L.H., A.R.W., N.C.N., A.S., V.B., and J.G. reviewed and edited the manuscript.

## Conflicts of Interest

The authors declare no conflicts of interest.

## Supporting information


Table S1.

## Data Availability

Data sharing not applicable to this article as no datasets were generated or analysed during the current study.

## Endnotes

^1^
We note there is no consensus on how to describe the TGD community broadly and use the term trans* to represent the multitude of trans identities including, but not limited to transgender, transsexual, transmasculine, and transfeminine people.
