# Supplementary material for: A Scoping Review Mapping Trans* and Gender Diverse People's Representation in Cancer Research
Source: Cancer Med. 2025 Aug 5;14(15):e70774. doi: 10.1002/cam4.70774 (PMC12322499; doi:10.1002/cam4.70774)
Supplement: Supplementary file 1 — Table S1. [file CAM4-14-e70774-s001.docx]

*Supplemental Table 1: Individual Study Results*

| **Author:**  **Last Name** | **Year** | **Location** | **Cancer type** | **Cancer continuum** | **Study design** | **Gender definition** | **TGD population (N)** | **Gender measure** | **Data source** |
| --- | --- | --- | --- | --- | --- | --- | --- | --- | --- |
| Asscheman | 2011 | Netherlands | Not specific | Mortality | Cohort | Not reported | Transsexual: Male-to-Female (MtF): 966 Female to Male (FtM): 365  All study participants were restricted to those using cross-sex hormones. | Determined based on being patient at Gender Clinic | Clinical Database from single institution - a specialized gender clinic  Routinely Collected Registry Data - Netherlands National Civil Record Registry for comparator |
| Luehmann | 2022 | USA | Breast | Screening | Not reported | Not reported | Transgender & Nonbinary people designated male at birth (TGNB DMAB): 193  Transgender & nonbinary people designated female at birth (TGNB DFAB): 60 | Key words identified in chart/clinical notes related to transgender and gender diverse people)  ICD codes and/or DSM diagnosis in chart | Clinical database from single institution |
| Bazzi | 2015 | USA | Breast | Screening | Not reported | Transgender: those whose gender identity is incongruent with their birth-assigned sex | Transgender  Transwomen: 28  Transmen: 13 | TGD-specific designations in EHR/registry data | Clinical database from single institution |
| Liu | 2021 | USA | Lung | Risk  Diagnosis  Treatment  Survival | Not reported | Transsexual or Transgender: people who desire, plan to undergo, or have undergone sex change surgery) | Gender Minority Individuals: 40-100 number suppressed  (not differentiated): | TGD-specific designations in EHR/registry data | Routinely Collected Registry Data - California Cancer Registry |
| Berner | 2021 | United Kingdom | Cervical | Screening | Cross-sectional | Transgender (trans) refers to people who do not identify with the gender assigned to them at birth. Trans men are birth- assigned females who identify as men or masculine, and non-binary is an umbrella term for the many trans people with gender identities that are neither exclusively male nor female. | Transmen & nonbinary people: 137  (not differentiated) | Self-reported | Participants completed a web-based survey |
| Oladeru | 2022 | USA | Breast  Cervical | Screening | Not reported | Not reported | Transgender:  MtF FtM  Breast cancer sample: 614 trans people  Cervical Cancer sample: 587 trans people | Self-reported | Behavioural Risk Factor Surveillance System: 2014-2016 & 2018 |
| Gooren | 2013 | Netherlands | Breast | Risk | Not reported | Transsexual people are those with apparently normal somatic sexual differentiation who strongly feel they actually belong to the opposite sex. | Transsexual: MtF: 2307 FtM: 795  All study participants were restricted to those using cross-sex hormones. | Determined based on being patient at Gender Clinic | Clinical database from single institution - a specialized gender clinic |
| de Blok | 2019 | Netherlands | Breast | Risk | Cohort | Transgender people experience an incongruence between the sex assigned to them at birth and their experienced or expressed gender. | Transgender: Trans woman: 2260 Trans men: 1229  All study participants were restricted to those using cross-sex hormones. | Determined based on being patient at Gender Clinic | Clinical database from single institution - a specialized gender clinic  Routinely collected registry data - Nationwide Network and Registry of Histopathology and Cytopathology in the Netherlands (PALGA)    Statistics Netherlands |
| Narayan | 2017 | USA | Breast | Screening | Not reported | Nonconforming refers to people who do not follow societal notions how they should look or act based on the sex they were assigned at birth | Transgender: MtF: 77 FtM: 112 Gender non-conforming: 31 | Self-reported | Behavioural Risk Factor Surveillance System: 2014 |
| Kiran | 2019 | Canada | Breast  Cervical  Colorectal | Screening | Cross-sectional | An umbrella term that includes but is not limited to people who identify as genderqueer, genderfluid, and gender nonbinary, and whose gender identities challenge societal gender norms. In this article, we use trans as an inclusive term for all of the above. Trans individuals have a gender identity or expression that is different than the sex that was assigned to them at birth. | Transgender: 120  Did not differentiate results but categorized transgender people on basis of sex assigned at birth - Assigned Male sex at birth & Assigned Female sex at birth | TGD-specific designations in EHR/registry data | Clinical database from single institution  Routinely collected registry data - Ontario cancer registry |
| Jackson | 2021 | USA | Not specific | Diagnosis  Treatment  Mortality | Not reported | Transgender is the umbrella term for a diverse group of individuals whose gen- der identity differs from their sex assigned at birth | Transgender: 589  (not differentiated) | TGD- specific designation in EHR/registry data  Note TGD designations using old terminology ie. transsexual or harmful language ie. hermaphrodite were reworded into more accepting/modern language | Routinely Collected registry data - National Cancer Database |
| Hutchinson | 2018 | USA | Not specific | Risk | Not reported | Not reported | Transgender: 230  (did not differentiate in results, but did note proportion of cases by natal sex) | TGD-specific designations in EHR/registry data Key words identified in chart related to transgender and gender diverse people) | Clinical database of hospitalization records across New York State  Routinely collected data - New York State Cancer Registry Hospital records linked to NYSCR |
| McDowell | 2017 | USA | Cervical | Screening | Not reported | Trans-masculine (TM) individuals have a masculine spectrum gender identity (men, male, trans man, trans male, nonbinary, or another diverse gender identity on the masculine continuum), but were assigned female sex at birth. | Transmasculine people:  31 interviews  32 survey respondents | Self-reported | Participants participated in interviews and completed online interviews |
| Maza | 2020 | El Salvador | Cervical | Screening | Cross-sectional | Transgender men - Men assigned female at birth | Transgender men: 24 | Self-reported | Transgender men completed a questionnaire and HPV self-sample swab |
| Silverberg | 2017 | USA | Not specific | Risk | Not reported | Not reported | Transgender people: Transmasculine: 2098 Transfeminine: 2791 | Key words identified in chart related to transgender and gender diverse people) ICD codes and/or DSM diagnosis in chart | Clinic database from three Kaiser Permanante sites  Routinely collected registry data - Surveillance Epidemiology and End Results' cancer registry |
| Davis | 2022 | USA | Cervical | Screening | Not reported | Female-to-male (FTM) transgender males are individuals who were assigned female gender at birth and currently identify within the masculine continuum. Their gender identity can include male, nonbinary, genderqueer, other, bigender, and agender. | Transgender: FtM: 89 | Key words identified in chart related to transgender and gender diverse people) ICD codes and/or DSM diagnosis in chart | Clinical database from single site |
| Meyer | 2017 | USA | Not specific | Risk | Not reported | Transgender people have a gender identity that differs from the sex assigned to them at birth. | Transgender and gender nonconforming: 691  (merged into transgender category in results) | Self-reported | BRFSS: 2014 |
| Stewart | 2020 | USA | Breast  Cervical | Screening | Not reported | Not reported | Transgender woman: 87 Transgender man: 146 Gender Nonbinary (GNB) + Gender Nonconforming + Genderqueer Gender diverse: 22   - AFAB – 13 - AMAB - 9 | Key words identified in chart related to transgender and gender diverse people   ICD codes and/or DSM diagnosis in chart or provider | Clinical database single institution |
| Burton | 2020 | United Kingdom | Not specific but focused on pelvic radiotherapy | Treatment | Survey | “Transgender” is an adjective used to describe a person for whom their gender does not match that which they were assigned at birth, based on their physical characteristics. A transgender man, for example, would have been assigned a female gender at birth. Some individuals may feel as though their gender cannot be described as “man” or “woman”. For some, this is because their gender ﬂuctuates; these individuals may use terms such as “genderﬂuid”. For others, they may identify with no gender at all, and prefer terms such as “agender” or “neutrois”. Others may simply feel that their gender identity exists outside of the man/woman dichotomy, and may use terms including “genderqueer” and “non- binary”. Non-binary is also often used as an umbrella term for all genders outside of man or woman. | Transmen & Nonbinary people:19 | Self-reported | Participants completed a web-based survey |
| Peitzmeier  (2014) | 2014 | USA | Cervical | Screeening | Not reported | Female-to-male (FTM) transgender individuals, also known as transgender men, are persons born with female reproductive organs who identify as male. | Transgender: FtM: 233 | Determined based on being patient at Gender Clinic | Clinical database from single institution |
| Nash | 2018 | USA | Not specific | Risk | Not reported | Transgender people comprise a diverse group of individuals whose biological sex does not match their gender identity. | Transgender: 805  (not differentiated) | TGD-specific designations in EHR/registry data  Note TGD designations using old terminology ie transsexual or harmful language ie hermaphrodite were reworded into more accepting/modern lanugage | Routinely Collected Registry Data - NAACCR from 46 states as well as DC for years between 1995-2013 |
| Tabaac | 2018 | USA | Breast  Cervical  Colorectal  Prostate | Screening | Not reported | Transgender is an umbrella term that includes transgender (i.e., individuals whose gender identity is different from their sex assigned at birth) men, women, and gender-nonconforming (i.e., individuals who do not identify solely as male or female; e.g., non-binary, genderqueer, agender, bigender, or gender- ﬂuid). | Transgender Men: 593 Transgender women: 936 Gender Non-conforming: 384 | Self-reported | BRFSS: 2014-2016 |
| Agénor  (2018) | 2018 | USA | Cervical | Screening | Not reported | Transmasculine individuals (i.e. individuals assigned female at birth who self-identify as men, transgender men, female-to-male [FtM], or another transmasculine gender identity | Transmasculine people: 122  Binary transmasculine people: 94 Non-binary transmasculine people: 28 | Self-reported | Participants completed self-administered survey |
| Goldstein | 2020 | USA | Cervical | Screening | Not reported | Transmasculine individuals (persons with masculine spectrum gender identity but recorded female sex at birth) | Transmasculine people:  314 transmasculine people -  121 baseline  193 intervention | Determined based on being patient at Gender Clinic | Clinical database from single institution |
| Kerr | 2022 | Australia | Cervical | Screening | Not reported | Trans and gender diverse population includes a diverse range of people who have genders that do not align with their sex assigned at birth and/or are outside of the binary man/woman. | Trans and gender diverse: 196  (not differentiated) | Self-reported | Participants completed a web-based survey |
| Brown GR | 2015 | USA | Breast | Risk | Not reported | Not reported | Transgender people:  5135  Results report natal sex of people observed to be diagnosed with breast cancer.  Further describes participants as FtM and MtF transsexuals. | ICD codes and/or DSM diagnosis in chart | Clinical database -Veterans Health Administrations  Routinely Collected Registry Data - SEER data |
| Fein | 2021 | USA | Anal | Screening | Not reported | Not reported | Transgender:  54 Transgender women  25 Transgender men | Self-reported | Participants completed a web-based survey |
| Stowell | 2020 | USA | Lung | Screening | Cross-sectional | Transgender and gender diverse persons (those whose gender identity does not align with the sex assigned to them at birth | Transgender: 143  (not differentiated)  Notably however, authors describe transgender binary and transgender non-conforming people, but collapse into one category | Self-reported | BRFSS: 2017 & 2018 |
| Weyers | 2010 | Belgium | Breast | Screening | Case control | Not reported | Transsexual women: 50 | Determined based on being patient at Gender Clinic | Clinical database: Single institution - specialized gender clinic |
| Charkhchi | 2019 | USA | Breast  Cervical  Colon | Screening | Not reported | Gender identity as one’s “innermost concept of self as male, female, a blend of both or neither—how individuals perceive themselves and what they call themselves,” which can be the same or different from that assigned at birth | Transgender:  Total population >40,000 MtF: 0.1% of population FtM: 0.14% of population Transgender Nonconforming: 0.06% of population | Self-reported | BRFSS: 2016 |
| Blosnich | 2014 | USA | Not specific | Mortality | Not reported | Transgender individuals include persons whose self-identiﬁed core gender identity (i.e., innate sense of self as a man, woman, neither, or both) is discordant with their assigned birth sex or the Western socially-constructed binary of masculine/feminine gender ascribed to birth sex. The term transgender is often used as an umbrella term which can include very diverse populations, including transsexual persons (persons who often choose medical and/or surgical treatments to transition from their birth sex to the opposite sex) and others with or without a diagnosis of gender dysphoria. | Transgender: 309  (not differentiated) | ICD codes and/or DSM diagnosis in chart | Clinical database Veterans Health Administrations  Routinely Collected Registry Data - National Death Index |
| de Blok | 2021 | Netherlands | Not specific | Mortality | Cohort | Transgender people, characterised by an incongruence between sex assigned at birth and gender identity | Transgender  Transgender women: 2927  Transgender men: 1641 | Determined based on being patient at Gender Clinic | Clinical database from single institution - a specialized gender clinic  Routinely Collected Registry Data: Statistics Netherlands |
| Lombardo | 2022 | USA | Not specific | Screening | Not reported | Not reported | FtM: 94  MtF: 75  Other: 40 | Self-reported | Participants completed a web-based survey |
| Wierckx | 2013 | Belgium | Not specific | Risk | Case control | Not reported | Transmen: 138  Transwomen: 214 | Determined based on being patient at Gender Clinic  ICD codes and/or DSM diagnosis in chart | Participants completed face- to-face interviews using a combination of computer- assisted personal interviewing and computer-assisted self-interviewing.  Clinical database from single institution - a specialized gender clinic |
| de Nie | 2020 | Netherlands | Prostate | Risk | Cohort | Transgender people experience an incongruence between the sex assigned at birth and their experienced or expressed gender | Transgender women: 2281 | Determined based on being patient at Gender Clinic | Clinical database from single institution - a specialized gender clinic  Routinely collected registry data - Nationwide Network and Registry of Histopathology and Cytopathology in the Netherlands (PALGA)    Statistics Netherlands |
| Ma | 2021 | USA | Prostate | Screening | Cross-sectional | Not reported | Transgender: MtF: 292 | Self-reported | BRFSS: 2014-2016 & 2018 |
| Pratt-Chapman | 2020 | USA | Breast  Cervical  Colorectal  Prostate  Lung  Anal  Skin | Screening | Cross-sectional | Not reported | Transgender & Gender nonconforming: 58  Further categorized by describing people as sex assigned at birth.brown  Results for each screening type included only eligible participants, i.e. people with cervix included in cervical screening. | Self-reported | Participants completed survey on electronic tablet at transgender-affirming community events. |
| Johnson | 2016 | USA | Cervical | Screening | Convergent Parallel | Not reported | FtM or Genderqueer:  15.5% of 226 | Self-reported | Participants completed web-based questionnaire and telephone interviews |
| Boehmer | 2020 | USA | Not specific | Risk  Survivorship | Cross-sectional | Transgender people are a diverse population comprised of individuals who reject a binary gender (eg, gender nonconforming) or who report a gender identity that is different from their assigned sex at birth. | Trans women: 1877 Trans men: 1344 Gender nonconforming: 876 | Self-reported | BRFSS: 2014-2018 (pooled) |
| Seay | 2017 | USA | Cervical | Screening | Not reported | Transgender men, or individuals assigned female sex at birth who self-identify as men | Transgender men: 91 | Self-reported | Participants completed a web-based survey |
| Grasso | 2020 | USA | Cervical | Screening | Not reported | Not reported | Transgender men: 99 Transgender women: 13 Other: 763 | TGD-specific designations in EHR/registry data | Clinical database from multiple institutions - 5 federally (US) qualified health centres |
| Kerr  2021 | 2021 | Australia | Not specific | Treatment | Phenomenological | TGD inclusive of people whose gender differs from sex assigned at birth | Trans and gender diverse people | Self-reported | Participants participated in interviews |
| Bryson | 2020 | Canada  USA | Breast  Gynecological | Treatment | Narrative | Not reported | Trans: 7  Gender nonconforming: 3 (as 'trans category)   Genderqueer: 38  (as non-trans identity) | Self-reported | Participants participated in interviews |
| Taylor | 2016 | Canada  USA | Breast  Gynecological | Treatment | Narrative | Trans* and gender nonconforming, where trans comes to stand in the place of "sex" or "gender" | Trans: 7  Gender nonconforming people: 3 | Self-reported | Participants participated in interviews |
| Peitzmeier | 2020 | USA | Cervical | Screening | Not reported | Transmasculine individuals are people assigned a female sex at birth (AFAB) who identify along a spectrum of masculinity or have a non-binary gender | Transmasculine people: 32 | Self-reported | Participants participated in interviews |
| Wentling | 2021 | USA | “Sex-specific”  Breast  Gynecological  Prostate | Screening | Not reported | The transgender population includes anyone whose gender identity does not match their birth sex assignment (Stryker, 2008). It includes people who identify as gender binary or gender nonbinary, and it is heterogeneous along a range of biological, psychological, and social dimensions. | Transgender people: 35 | Self-reported | Participants participated in interviews |
| Alpert | 2021 | USA | Not specific | Treatment | Interpretive description | Not reported | Transgender: 7 | Self-reported | Participants participated in group interviews |
| Peitzmeier | 2017 | USA | Cervical | Screening | Not reported | Transmasculine individuals assigned a female sex at birth and whose gender identity lies on a continuum of masculinity or gender nonconformity (e.g., female-to-male, transgender man, male, or genderqueer | Transmasculine people: 32 | Self-reported | Participants participated in interviews |
| Ussher | 2022 | Primarily Australia other countries included:  USA  United Kingdom  New Zealand  Canada | Not specific | Treatment | Not reported | Gender identity as one’s “innermost concept of self as male, female, a blend of both or neither—how individuals perceive themselves and what they call themselves,” which can be the same or different from that assigned at birth | Transgender and gender diverse  Patients –  Trans female: 13  Trans male: 8  Non-binary: 34  Other/multiple identities: 14  Carers –  Trans female: 5  Trans male: 2  Non-binary: 16 | Self-reported | Participants completed survey and participated in interviews |
| Agénor | 2016 | USA | Cervical | Screening | Not reported | Transmasculine individuals (i.e., people assigned female sex at birth who self-identify as men, male, transgender men, female-to-male or a non-binary gender identity along the transmasculine continuum | Transmasculine people: 32 | Self-reported | Participants participated in interviews |
| Johnson | 2020 | USA | Cervical | Screening | Exploratory | TM are individuals whose gender identity does not match with their natal female sex. For the purposes of this paper, TM is an umbrella term that refers to any natal female sex person who identiﬁes with a diﬀerent gender. | Transgender Men: 20 | Self-reported | Participants participated in interviews |
| Brown MT | 2018 | USA | Breast | Treatment | Cross-sectional | Not reported | Genderqueer or transgender: 68  (not differentiated) | Self-reported | Participants completed web-based survey |
| Power | 2022 | Australia  USA  United Kingdom  New Zealand  Canada | Not specific | Treatment  Survivorship | Not reported | Not reported | Trans (binary and non-binary): 63  Different gender identity: 6 | Self-reported | Participants completed survey, participated in interviews, and photovoice |
| Carr | 2018 | USA | Not specific | Treatment | Not reported | Not reported | Gender non-binary: 1 | Self-reported | Participants completed a survey interview |
| Kamen | 2018 | USA | Not specific | Treatment | Not reported | Not reported | Transgender woman: 2 Transgender man: 7  Other: 2 | Self-reported | Participants completed a web-based survey |
